# Novel Eu^3+^-Doped Glasses in the MoO_3_-WO_3_-La_2_O_3_-B_2_O_3_ System: Preparation, Structure and Photoluminescent Properties

**DOI:** 10.3390/molecules29194687

**Published:** 2024-10-03

**Authors:** Lyubomir Aleksandrov, Margarita Milanova, Aneliya Yordanova, Reni Iordanova, Kenji Shinozaki, Tsuyoshi Honma, Takayuki Komatsu

**Affiliations:** 1 Institute of General and Inorganic Chemistry, Bulgarian Academy of Sciences, G. Bonchev, str. bld. 11, 1113 Sofia, Bulgaria; lubomirivov@gmail.com (L.A.); a.yordanova@svr.igic.bas.bg (A.Y.); reni@svr.igic.bas.bg (R.I.); 2 National Institute of Advanced Industrial Science and Technology (AIST), 1-8-31 Midorigaoka, Ikeda, Osaka 563-8577, Japan; k-shinozaki@aist.go.jp; 3 Department of Materials Science and Technology, Nagaoka University of Technology, 1603-1 Kamitomioka-cho, Nagaoka 940-2188, Japan; honma@mst.nagaokaut.ac.jp (T.H.); komatsu@mst.nagaokaut.ac.jp (T.K.)

**Keywords:** glasses, structure, europium, photoluminescence, red-emitting glass materials

## Abstract

Novel multicomponent glasses with nominal compositions of (50−x)MoO_3_:xWO_3_:25La_2_O_3_:25B_2_O_3_, x = 0, 10, 20, 30, 40, 50 mol% doped with 3 mol % Eu_2_O_3_ were prepared using a conventional melt-quenching method. Their structure, thermal behavior and luminescent properties were investigated by Raman spectroscopy, differential thermal analysis and photoluminescence spectroscopy. The optical properties of the glasses were investigated by UV–vis absorption spectroscopy and a determination of the refractive index. Physical parameters such as density, molar volume, oxygen molar volume and oxygen packing density were determined. The glasses are characterized by a high glass transition temperature. Raman analysis revealed that the glass structure is built up mainly from tetrahedral (MoO_4_)^2−^ and (WO_4_)^2−^ units providing Raman bands of around 317 cm^−1^, 341–352 cm^−1^, 832–820 cm^−1^ and 928–935 cm^−1^. At the same time, with the replacement of MoO_3_ with WO_3_ some fraction of WO_6_ octahedra are produced, the number of which increases with the increasing WO_3_ content. A strong red emission from the ^5^D_0_ level of Eu^3+^ ions was registered under near-UV (397 nm) excitation using the ^7^F_0_ → ^5^L_6_ transition of Eu^3+^. Photoluminescence (PL) emission gradually increases with increasing WO_3_ content, evidencing that WO_3_ is a more appropriate component than MoO_3_. The integrated fluorescence intensity ratio R (^5^D_0_ → ^7^F_2_/^5^D_0_ → ^7^F_1_) was calculated to estimate the degree of asymmetry around the active ion, suggesting a location of Eu^3+^ in non-centrosymmetric sites. All findings suggest that the investigated glasses are potential candidates for red light-emitting phosphors.

## 1. Introduction

Trivalent europium-doped materials are usually considered as good red-emitting phosphor candidates for LEDs. Its characteristic energy transfer generates a strong emission with a high color purity [1]. Unfortunately, Eu^3+−^-doped materials cannot be efficiently excited by present LED chips, because their excitation peaks are weak in nature due to parity-forbidden ff transitions [2]. The use of inorganic host matrices with strong absorption in the ultraviolet (UV) region, which occurs commonly via excitation under ligand-to-metal charge transfer (LMCT) absorption bands, is a usual approach to improve the luminescence intensities of the Eu^3+^ materials [3]. Among many inorganic compounds, molybdates and tungstate phases have been widely studied for decades as hosts for lanthanide doping due to their absorption in the mid-ultraviolet region via O(2p) → W(5d)/Mo(4d) charge transfer and the subsequent transfer of energy to the low-lying emissive states of trivalent lanthanide ions [3,4]. At present, reports are mainly focused on the preparation of the crystalline molybdate and tungstate host structures, while data for the molybdate and tungstate glass and glass-ceramic rare-earth hosts are limited [1,2,3,4,5,6,7,8,9,10,11,12,13,14,15,16]. Compared with bulk crystalline hosts, glasses have the advantages of easy fabrication, low cost, high mechanical strength and high chemical durability. Therefore, it is meaningful to prepare molybdate and tungstate glass compositions doped with rare-earth activators and to investigate their luminescence.

In our previous works, we have reported the preparation of Eu^3+^-doped glasses and glass-ceramics with a high WO_3_ content in the systems WO_3_-La_2_O_3_-B_2_O_3_ and WO_3_-La_2_O_3_-B_2_O_3_-Nb_2_O_5_ possessing strong 613 nm red luminescence with excitation at 390 nm, an indication that they could be promising materials for emitting red light. [17,18]. In our more recent works, we have obtained tungsten-containing ZnO–B_2_O_3_ glasses doped with Eu^3+^ active ions and we have studied their luminescent properties [19,20,21]. The obtained results pertaining to glass structure, physical, thermal and optical properties indicate the suitability of the 50ZnO:40B_2_O_3_:10WO_3_ glass network for the luminescence performance of Eu^3+^ ions. The positive effect of the addition of WO_3_ on luminescence intensity is proven by the stronger Eu^3+^ emission of the zinc–borate glass containing WO_3_ compared to the WO_3_-free zinc–borate glass, a phenomenon engendered mainly by the energy transfer from tungstate groups to the Eu^3+^ ions (sensitizing effect). The most intense luminescence peak observed at 612 nm and the high-integrated emission intensity ratio (R) of the ^5^D_0_ → ^7^F_2_/^5^D_0_ → ^7^F_1_ transitions at 612 nm and 590 nm of 5.77 suggest that the glasses have potential as red light emission materials. We have also prepared homogeneous, optically transparent ternary MoO_3_-La_2_O_3_-B_2_O_3_ and WO_3_-La_2_O_3_-B_2_O_3_ glasses containing a large amount of MoO_3_ (10–50 mol%) and WO_3_ (15–50 mol%) as well as quaternary glasses with nominal compositions of (50−x)MoO_3_:xWO_3_:25La_2_O_3_:25B_2_O_3_, x = 0, 10, 20, 30, 40, 50 mol% and we have investigated their structure and crystallization behavior [22,23,24]. It was proposed that the glass structure of ternary and quaternary glasses is built up mainly from tetrahedral (MoO_4_)^2−^ and (WO_4_)^2−^ units and BO_3_ and BO_4_ groups. The main crystalline phases in the crystallized MoO_3_-La_2_O_3_-B_2_O_3_ glasses were found to be LaMoBO_6_ and LaB_3_O_6_. The formation of LaMo_x−1_W_x_O_6_ solid solutions was confirmed in the crystallized samples from the MoO_3_-WO_3_-La_2_O_3_-B_2_O_3_ system.

In this paper, we continue our investigations into glasses in the MoO_3_-WO_3_-La_2_O_3_-B_2_O_3_ system. The purpose is to obtain Eu^3+^-doped glasses with nominal compositions of (50−x)MoO_3_:xWO_3_:25La_2_O_3_:25B_2_O_3_:3Eu_2_O_3_, x = 0, 10, 20, 30, 40, 50 mol% and to study their thermal behavior, structure and luminescent properties.

## 2. Results and Discussion

### 2.1. Thermal Properties

**Figure 1:**
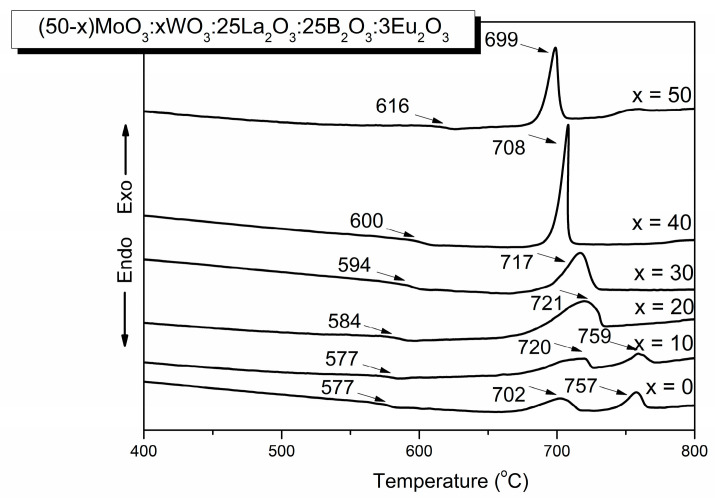
DTA curves of (50−x)MoO_3_:xWO_3_:25La_2_O_3_:25B_2_O_3_:3Eu_2_O_3_ x = 0, 10, 20, 30, 40, 50 mol% glasses.

The amorphous nature of the obtained materials has been proved by differential thermal analysis (DTA). The DTA curves of the (50−x)MoO_3_:xWO_3_:25La_2_O_3_:25B_2_O_3_:3Eu_2_O_3_, x = 0, 10, 20, 30, 40, 50 mol% glasses are shown in Figure 1. The endothermic dips corresponding to the glass transition temperature (Tg), and the exothermic peaks due to the crystallization temperature (Tc) are observed. The estimated values of Tg and Tc are pointed out in the figure. As it is seen, the glass transition temperature increases from 577 °C to 616 °C with the substitution of WO_3_ for MoO_3_ because of the replacement of weaker Mo–O bonds with stronger W–O bonds [24]. In the DTA curves of glasses with a higher MoO_3_ content (x = 0 and x = 10), two broad exothermic peaks are observed, while glasses with a higher WO_3_ content (x = 50 and x = 40) are characterized with one sharp and intensive exothermic effect, evidencing different crystallization behaviors depending on composition. The x = 0 glass has higher thermal stability against crystallization, i.e., ΔT = Tc − Tg = 125 °C compared to the ΔT of x = 50 glass (83 °C), evidencing the better glass-forming ability of molybdate compared to tungstate glass.

On the other hand, glasses containing a higher amount of MoO_3_ and lower WO_3_ content up to 20 mol% have the highest ΔT values (145 °C for glass x = 10 and 137 °C for glass x = 20), indicating that the addition of a small amount of WO_3_ into the molybdate glass improves the glass formation tendencies of the compositions.

### 2.2. Structural Investigations

#### 2.2.1. Raman Analysis

**Figure 2:**
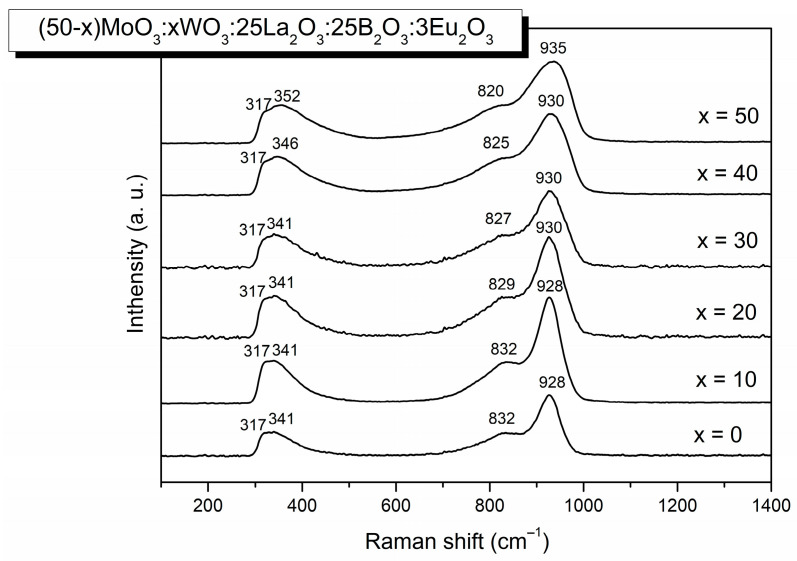
Raman spectra of (50−x)MoO_3_:xWO_3_:25La_2_O_3_:25B_2_O_3_:3Eu_2_O_3_, x = 0, 10, 20, 30, 40, 50 mol% glasses.

Raman spectra of (50−x) MoO_3_:xWO_3_:25La_2_O_5_:25B_2_O_3_:3Eu_2_O_3_ (x = 0, 10, 20, 30, 40, 50 mol%) glasses are shown in Figure 2. All spectra consist of broad Raman bands at around 317 cm^−1^, 341–352 cm^−1^, 832–820 cm^−1^ and 928–935 cm^−1^. The spectra obtained are similar to the spectra of four component glasses (50−x)MoO_3_:xWO_3_:25La_2_O_5_:25B_2_O_3_, x = 10, 20, 30, 40, 50 and three component xMoO_3_:25La_2_O_3_:(75−x)B_2_O_3_, x = 10–50 and xWO_3_:25La_2_O_3_:(75−x)B_2_O_3_, x = 15, 25, 50 glasses previously reported and discussed by us in detail [22,23,24].

Based on these earlier data, and bearing in mind spectral findings for other molybdate and tungstate glass and crystalline phases [25,26,27], we can assign the Raman bands obtained as follows. The most intense band at 928–935 cm^−1^ is ascribed to the symmetric stretching vibration mode ν_1_ of isolated (MoO_4_)^2−^ and (WO_4_)^2−^ tetrahedral units. As seen in Figure 2, this band becomes broader with an increase in WO_3_ content. Spectral deconvolution performed in [22,23,24] has shown the presence of a weak band at 980–996 cm^−1^ related to WO_6_ octahedral groups in WO_3_-containing glasses. The band at 832–820 cm^−1^ is due to the asymmetric ν_3_ stretching vibration of (MoO_4_)^2−^ and (WO_4_)^2−^ groups. Two Raman bands in the low-frequency spectral region of 315 cm^−1^ and 341–352 cm^−1^ are attributed to the overlapping ν_2_ and ν_4_ vibrations of [MoO_4_]^2−^ and [WO_4_]^2−^ tetrahedra, with lower values in both bands corresponding to [MoO_4_]^2−^ tetrahedra. The band at 341–352 cm^−1^ might contain some contribution of the vibration of LaO_n_ polyhedra in the glasses, as it is reported in the literature that La_2_O_3_ produces Raman bands below 400 cm^−1^ [25]. At the same time, Eu-O vibrations are expected at ~315 cm^−1^ [20]. In the Raman spectra obtained, there are no peaks in the region of 1000–1500 cm^−1^, where Raman bands of the boron–oxygen groups are situated [20]. However, bearing in mind our previous works on similar glass compositions [22,23,24], it could be suggested that BO_3_ and BO_4_ groups and B–O–B bonds are also present in the structure of the investigated glasses. The spectral results obtained suggest that the structure of (50−x)MoO_3_:xWO_3_:25La_2_O_5_:25B_2_O_3_:3Eu_2_O_3_ (x = 0, 10, 20, 30, 40, 50 mol%) glasses consists of mainly (MoO_4_)^2−^ and (WO_4_)^2−^ tetrahedral units, the fraction of which changes continuously with the substitution of WO_3_ for MoO_3_. At the same time, with the replacement of MoO_3_ with WO_3_ some amount of WO_6_ octahedra are produced, the number of which increases with the increasing WO_3_ content.

#### 2.2.2. Physical Parameters

Structural information of the glasses was also gained by density (ρ_g_) measurements which provided the base for several values of physical parameters such as: molar volume (V_m_), oxygen molar volume (V_o_) and oxygen packing density (OPD). These were evaluated using the following relations:

(1) $$V_{m}=\frac{\sum x_{i}M_{i}}{\rho_{g}}$$

(2) $$V_{o}=V_{m}\times \left(\frac{1}{\sum x_{i}n_{i}}\right)$$

(3) $$OPD=1000\times C\times \left(\frac{\rho_{g}}{M}\right)$$

where x_i_ is the molar fraction of each component i, M_i_ is the molecular weight, ρ_g_ is the glass density, n_i_ is the number of oxygen atoms in each oxide, C is the number of oxygen atoms per formula units, and M is the total molecular weight of the glass compositions. The values obtained are listed in Table 1. As seen from the table, the density increases with the increasing WO_3_ content at the expense of MoO_3_ because of the replacement of lighter MoO_3_ (molecular weight 143.94 g/mol) with heavier WO_3_ (molecular weight 231.84 g/mol). The V_m_ and V_o_ values of the glasses decrease, while their OPD values become greater with the gradual replacement of MoO_3_ with WO_3_, evidencing better packing and bonding in the glass network with the introduction of WO_3_ [28].

It was found in our earlier paper that WO_6_ and W-O-W bridging bonds are formed in the glass network of (50−x)MoO_3_:xWO_3_:25La_2_O_3_:25B_2_O_3_, x = 10, 20, 30, 40, 50 and xWO_3_:25La_2_O_3_:(75−x)B_2_O_3_, x = 15, 25, 50 glasses [23,24]. The presence of bridging oxygens generates a more connected glass structure, resulting in the observed increase in density and OPD and a decrease in molar volume. The almost linear relationship between the density, established physical parameters and WO_3_ content suggests an increasing number of WO_6_ and their gradual polymerization (i.e., formation of W-O-W bonds) with WO_3_ loading.

#### 2.2.3. Optical Properties

**Figure 3:**
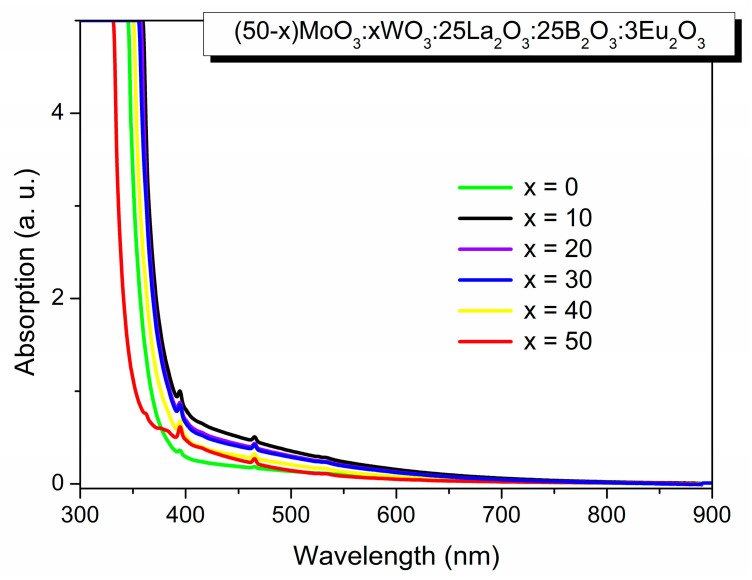
Optical absorption spectra at room temperature of (50−x)MoO_3_:xWO_3_:25La_2_O_3_:25B_2_O_3_-3Eu_2_O_3_, x = 0, 10, 20, 30, 40, 50 mol% glasses.

The optical absorption spectra at room temperature of (50−x) MoO_3_:xWO_3_:25La_2_O_5_:25B_2_O_3_:3Eu_2_O_3_ (x = 0, 10, 20, 30, 40, 50 mol%) glasses are shown in Figure 3. The absorption edge of ternary glasses, containing only MoO_3_ or WO_3_ (x = 0 and x = 50, respectively) shifts to the lower wavelength value in the UV range as compared with glasses containing both MoO_3_ and WO_3_. For instance, the absorption edge of glass x = 50 is 334.7 nm, while for x = 10 the value of the absorption edge shifts to 364.2 nm.

The refractive index (n) for the presented glasses is also established. It is found that the refractive index increases with the increasing WO_3_ content indicating the more densely packed structure in the presence of tungsten [29].

**Figure 4:**
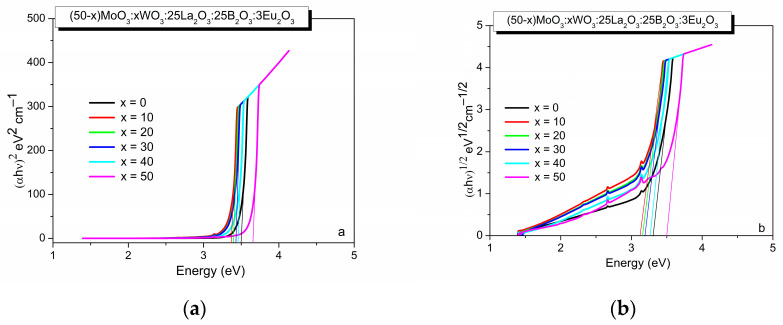
Tauc plot of (50−x)MoO_3_:xWO_3_:25La_2_O_3_:25B_2_O_3_:3Eu_2_O_3_, x = 0, 10, 20, 30, 40, 50 mol% glasses: (**a**) for direct transition, (**b**) for indirect transition.

Some structural information also can be obtained from the optical band gap values (E_g_) evaluated from the UV–vis spectra with the Tauc method by plotting (F(R_∞_) hν)^n^, where n = 0.5 or 2 for direct or indirect transition versus hν (incident photon energy), as shown in Figure 4a,b and in Table 1 [30]. As seen from Table 1, the E_g_ values of glasses containing both MoO_3_ and WO_3_, increases with the increasing WO_3_ content due to an increase of the bridging W–O–W bond concentration as a result of the accumulation of WO_6_ structural units and their gradual polymerization [31]. This result coincides well with the variation in the physical parameters established. On the other hand, the E_g_ value of the molybdate glass x = 0 is lower than that of the tungstate glass x = 50, evidencing the higher number of non-bridging oxygen species in the structure of the glass x = 0 since it is accepted that, in metal oxides, the creation of non-bonding orbitals with higher energy than bonding values shifts the valence band to higher energy, which results in E_g_ decreasing [32].

To summarize this section, the Raman data obtained and as well as the established values of the structurally sensitive physical parameters demonstrate that the structure of (50−x) MoO_3_:xWO_3_:25La_2_O_5_:25B_2_O_3_:3Eu_2_O_3_ (x = 0, 10, 20, 30, 40, 50 mol%) glasses consists of mainly (MoO_4_)^2−^ and (WO_4_)^2−^ tetrahedral units, the fraction of which changes continuously with the substitution of WO_3_ for MoO_3_. BO_3_, BO_4_ groups and B–O–B bonds also exist in the glass networks. Also, with the replacement of MoO_3_ with WO_3_ some amount of WO_6_ octahedra are produced, the number of which increases with the increasing WO_3_ content. The WO_6_ octahedra gradually polymerize forming W–O–W bonds with the increasing WO_3_ content. Thus, with the substitution of MoO_3_ with WO_3_ more disordered and connected glass structure is formed which is favorable for doping with Eu^3+^ active ions. DTA analysis also shows the increasing values of glass transition temperatures with the increasing WO_3_ concentration confirming the formation of more connected and stable glass networks.

##### 2.3. Luminescent Properties

**Figure 5:**
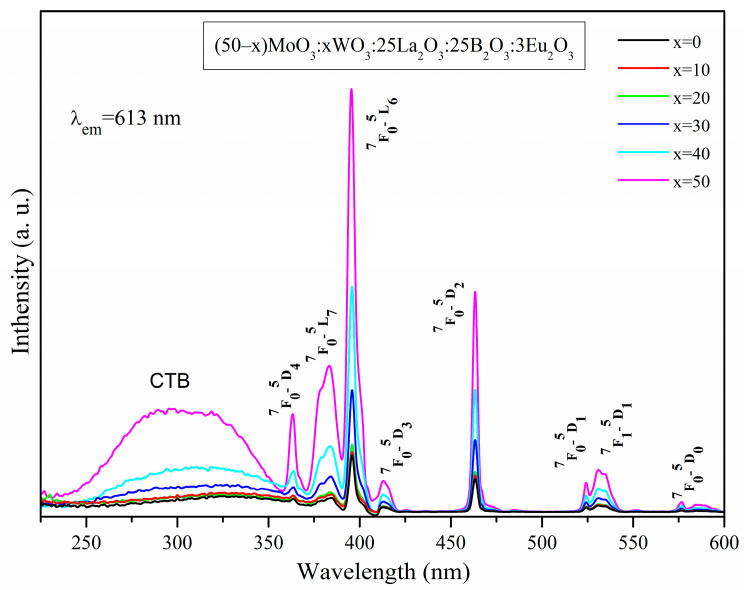
Excitation spectra of (50−x)MoO_3_:xWO_3_:25La_2_O_3_:25B_2_O_3_:3Eu_2_O_3_ (x = 0, 10, 20, 30, 40 and 50 mol%) glasses.

The photoluminescence excitation (PLE) spectra of the Eu^3+^-doped glasses are displayed in Figure 5. All data were obtained at room temperature by monitoring the most intensive characteristic emission of Eu^3+^ ions at a 613 nm wavelength, corresponding to ^5^D_0_ → ^7^F_2_ transition. From Figure 5, it can be observed that the PLE spectra consist of a broad continuous band ranging from 220 to 350 nm and narrow peaks in the 350–600 nm wavelength range. Generally, the excitation broadband in the ultraviolet region arises due to the ligand-to-metal charge transfer (LMCT) from O^2−^ ligand to W^6+^/Mo^6+^ ions in WO_n_ groups (WO_n_ = WO_4_ and WO_6_) and MoO_n_ groups (MoO_n_ = MoO_4_) of the glass matrix as well as from O^2–^ ions to Eu^3+^ ions, i.e., electron transfer from the 2p orbital of O^2−^ to the empty 4f orbital of Eu^3+^ [4,33]. As seen in Figure 5, in the PLE spectrum of the glass x = 50, two maxima of the LMCT band are observed—one at about 260 nm and the other at about 325 nm. Considering the previous assignments of PLE peaks of Eu^3+^ ions [34,35,36,37], the bands at around 260 nm and 325 nm in the excitation spectra obtained would be assigned mainly to the O^2−^ → Eu^3+^ and O^2−^ → W^6+^/Mo^6+^ LMCT transitions, respectively. The stronger intensity of the band at around 260 nm compared to the band at around 325 nm suggests that the O^2−^ → Eu^3+^ LMCT is taking place largely in the glass x = 50.

The presence of the excitation band of MoO_n_ and WO_n_ groups, recorded at the emission wavelength of Eu^3+^ at 613 nm suggests the existence of non-radiative energy transfer from the glass matrix to the active rare-earth ion [4,38]. As can be seen, the intensity of this band highly depends on the WO_3_ concentration and increases with the increase of WO_3_ content, suggesting that the energy transitions O^2−^ → W^6+^, in comparison to O^2−^ → Mo^6+^, largely influence the intensity of the charge transfer absorption band of the host matrix. Thus, it can be assumed that WO_3_ will contribute predominantly to the non-radiative energy transfer to the Eu^3+^ active ions and that the glasses containing significant tungsten oxide concentrations will exhibit the most intense emissions. This process is known as host-sensitized luminescence. 

The excitation spectra also show several peaks in the 350–600 nm wavelength range, assigned to the ff intra-configurational forbidden transitions of Eu^3+^ from the ground state (^7^F_0_) and from the first excited state (^7^F_1_): ^7^F_0_ → ^5^D_4_ (363 nm), ^7^F_1_ → ^5^L_7_ (383 nm), ^7^F_0_ → ^5^L_6_ (395 nm), ^7^F_0_ → ^5^D_3_ (412 nm), ^7^F_0_ → ^5^D_2_ (463 nm), ^7^F_0_ → ^5^D_1_ (523 nm), ^7^F_1_ → ^5^D_1_ (531 nm) and ^7^F_0_ → ^5^D_0_ (577 nm) 39], of which the ^7^F_0_ → ^5^L_6_ (397 nm) transition is the most intensive and was considered as an excitation wavelength to record the emission spectra. Compared to the LMCT, the ff transitions are stronger and their intensity increases as the concentration of WO_3_ increases, which is advantageous for achieving appropriate excitation by near-UV and blue LED chips, since in general the intensity of these Eu^3+^ transitions is weak due to the fact that they are forbidden by Laporte`s selection rule [39].

**Figure 6:**
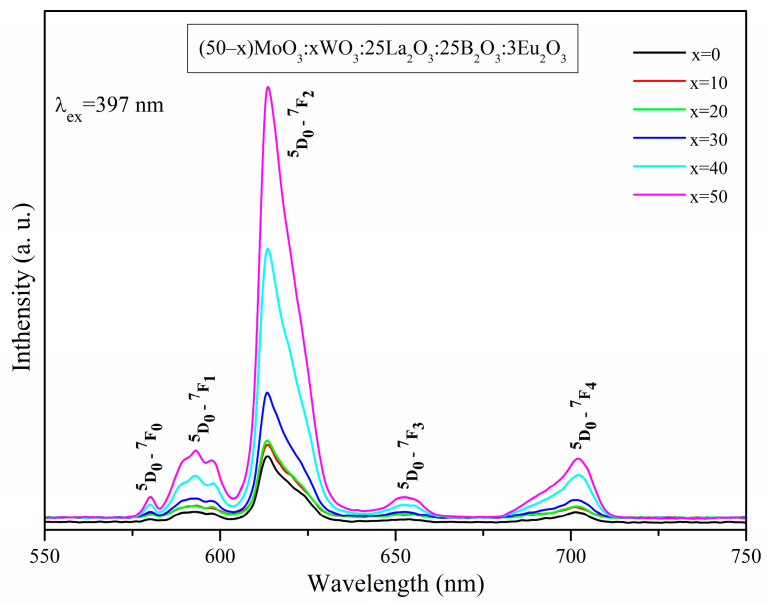
Emission spectra of (50−x)MoO_3_:xWO_3_:25La_2_O_3_:25B_2_O3:3Eu_2_O_3_ (x = 0, 10, 20, 30, 40 and 50 mol%) glasses.

The photoluminescence emission (PL) spectra of Eu^3+^-doped glasses, recorded under the most intensive Eu^3+^ excitation at 397 nm light, are shown in Figure 6. The characteristic emission peaks originating from the radiative transitions of Eu^3+^ ions from the ^5^D_0_ excited state to the lower-lying ^7^F_0_, ^7^F_1_, ^7^F_2_, ^7^F_3_, ^7^F_4_ ground states are observed at 580 nm, 593 nm, 613 nm, 652 nm and 702 nm, respectively. The dominant luminescent band is located at 613 nm. From Figure 6, it is clear that the emission intensity strongly depends on the WO_3_ concentration and increases considerably as its content increases. This observation may be due to the occurring non-radiative charge transfer from the glass host to the active Eu^3+^ ion. Evidence for the existence of the energy transfer is the absence of the characteristic broad emissions of WO_3_ and MoO_3_ in the spectral range 400–600 nm [40,41,42] arising from the fact that the energy absorbed by the tungstate and molybdate groups has transferred non-radiatively to the active Eu^3+^ ion.

Among all the observed emission transitions, the ^5^D_0_ → ^7^F_2_ transition is identified as electric dipole (ED) and is forced by the crystal field environment in the vicinity of the Eu^3+^ ions, while the ^5^D_0_ → ^7^F_1_ transition is magnetic dipole (MD) in nature, independent of the host matrix.

When Eu^3+^ ions are embedded in sites with inversion symmetry, the ^5^D_0_ → ^7^F_1_ magnetic dipole transition will dominate; on the contrary, when a Eu^3+^ ion site is non-centrosymmetric, the ^5^D_0_ → ^7^F_2_ electric dipole transitions will be the strongest in terms of emission. As a result, the luminescence intensity ratio (R) between electric (^5^D_0_ → ^7^F_2_) and magnetic (^5^D_0_ → ^7^F_1_) dipole transitions can be used as a probe into the nature of the site and symmetry of the coordination sphere, providing valuable information about the local symmetry around the rare-earth ion, the strength of covalent bonding between Eu^3+^ and its surrounding ligands and the emission intensity. The higher the value of R, the more distortion from inversion symmetry is observed, as well as higher covalency between Eu^3+^ and O^2−^ ions and increased emission intensity [11,43,44]. The calculated intensity ratios R (7.09–7.82) of the obtained glasses (Table 2) are higher than those of glasses previously synthesized by us [20,45,46,47] and those of other Eu^3+^-doped oxide glasses reported in the literature [16,48,49,50,51,52,53], as well as the commercially available red phosphors Eu^3+^:Y_2_O_3_ [54,55] and Eu^3+^:Y_2_O_2_S [56], suggesting that the synthesized samples are characterized by greater asymmetry in the vicinity of Eu^3+^ ions, stronger Eu-O covalence and an enhanced emission intensity. From Table 2, it can also be seen that the value of asymmetric ratio is increasing as the WO_3_ content increases and, as a result, stronger luminescence is observed.

Additional evidence of the low site symmetry in the vicinity of the active Eu^3+^ ions is the presence of the ^5^D_0_ → ^7^F_0_ transition, which is strictly forbidden and, according by Binnemans, appears in emission spectra when Eu^3+^ ions are located in sites with C_2v_, C_n_ or C_s_ symmetry [43]. To further examine the symmetry of the Eu^3+^ sites, the ^5^D_0_ → ^7^F_1_ transition is considered, which displays splitting. This implies that the symmetry of the Eu^3+^ sites in the studied glasses are C_2v_ or lower [57].

The observed optical properties are discussed on the basis of the glasses’ structural features. The most intensive Eu^3+^ emission peak, corresponding to the hypersensitive ^5^D_0_ → ^7^F_2_ transition, along with the high values of the luminescent ratio R, evidence that Eu^3+^ ions are located in low site symmetry in the host matrix. This emission peak intensity, and the increase in the R values of investigated glasses with the increasing concentration of WO_3_, indicates that the substitution of MoO_3_ with WO_3_ contributes to the creation of a more distorted and rigid glass structure that lowers the site symmetry of the rare-earth ion and improves its photoluminescence behavior. Furthermore, the increasing intensity of the band at 613 nm (^5^D_0_ → ^7^F_2_ transition) along with the increasing WO_3_ concentration indicates that the WO_3_ contributes predominantly to the non-radiative energy transfer to the Eu^3+^ active ions (host-sensitized luminescence). Thus, WO_3_ is a more appropriate component than MoO_3_ for enhancing the luminescent intensity of the doped Eu^3+^ ion.

## 3. Materials and Methods

The glasses with the nominal compositions of (50−x)MoO_3_:xWO_3_:25La_2_O_3_:25B_2_O_3_:3Eu_2_O_3_, x = 10, 20, 30, 40, 50 (mol%) were prepared using a conventional melt-quenching method. Reagent-grade commercial powders of MoO_3_, WO_3_, La_2_O_3_, Eu_2_O_3_ and H_3_BO_3_ were used as starting materials and were mixed in an alumina mortar. The batches (each batch weight: 10 g) were melted at 1200–1250 °C for 30 min in a platinum crucible in air. The glasses were obtained by pouring the melts onto an iron plate and by pressing with another iron plate (cooling rate ~10 K/s). The glass transition (T_g_) and crystallization peak (T_c_) temperatures were determined using differential thermal analysis (DTA) (Rigaki Thermo Plus TG 8120) at a heating rate of 10 K/min (±1). Optical absorption spectra of the glasses at room temperature were measured in the wavelength range of 200–800 nm using a spectrometer (Shimadzu U-3120). The uncertainty in the observed wavelength is about ±1 nm. Refractive indices at a wavelength (λ) of 632.8 nm (He–Ne laser) were measured at room temperature with a prism coupler (Metricon Model 2010). Densities of the glasses at room temperature were determined with the Archimedes method using distilled water as an immersion liquid in which measurements were repeated five times and the average value was used (±0.001g/cm^3^). Raman scattering spectra at room temperature were measured with a laser microscope (Tokyi Instruments Co. Nanofinder) operated at Ar^+^ (λ = 448 nm) with a laser resolution of ±1 cm^−1^. PL spectra of the glass samples at room temperature were measured with a PL spectrometer (JASCO FP-6500).

## 4. Conclusions

Glasses with a nominal composition of (50−x)MoO_3_:xWO_3_:25La_2_O_3_:25B_2_O_3_:3Eu_2_O_3_ (x = 0, 10, 20, 30, 40 and 50 mol%) were synthesized by a melt-quenching method and their structure, thermal behavior and luminescent properties were studied. The glass transition temperature increases with the substitution of MoO_3_ with WO_3_. Glasses containing a higher amount of MoO_3_ and a lower WO_3_ content, up to 20 mol%, have the highest thermal stability (ΔT = 145 °C for glass x = 10 and ΔT = 137 °C for glass x = 20). On the basis of Raman analysis, as well as density measurements and values of structurally sensitive physical parameters, it was established that the glass structure is built up mainly from (MoO_4_)^2−^ and (WO_4_)^2−^ tetrahedral units. WO_6_ octahedral groups and W-O-W bonds are also formed in the WO_3_-containing glasses, increasing in number with an increase of WO_3_ content. By UV-vis absorption spectroscopy, it was found that the obtained glasses were characterized by a transmittance above 334.7 nm. Glasses also possess a high refractive index between 1.93144 and 1.97066 depending on their composition. The luminescent properties of the obtained Eu^3+^-doped glasses revealed that they could be excited by 397 nm and exhibit pure red emission centered at 613 nm (^5^D_0_ → ^7^F_2_ transition). The Eu^3+^ luminescent intensity was found to increase with the WO_3_ loading. All findings obtained here are favorable for the elaboration of novel red-emitting glass materials.

## Figures and Tables

**Table 1:** Values of the physical parameters of glasses (50−x)MoO_3_:xWO_3_:25La_2_O_3_:25B_2_O_3_:3Eu_2_O_3_, x = 10, 20, 30, 40, 50 mol%: density (ρ_g_), molar volume (V_m_), oxygen molar volume (V_o_), oxygen packing density (OPD), optical band gap (E_g_), absorption edge A, refractive index, n.

Sample ID	ρ_g_ (±0.01) (g/cm^3^)	V_m_ (cm^3^/mol)	V_o_ (cm^3^/mol)	OPD (g atom/L)	E_g_ Direct (eV)	E_g_ Indirect (eV)	A (nm)	Refractive Index, n (Wavelength of 632.8 nm)
x = 0	4.756	38.14	12.34	81.02	3.50	3.31	350.7	1.93144
x = 10	4.992	38.10	12.33	81.10	3.37	3.12	367.2	1.93452
x = 20	5.439	36.58	11.84	84.47	3.39	3.16	364.1	1.94278
x = 30	5.729	36.26	11.73	85.22	3.43	3.19	359.8	1.95236
x = 40	6.064	35.71	11.56	86.53	3.46	3.27	353.7	1.96115
x = 50	6.403	35.19	11.39	87.81	3.66	3.50	334.7	1.97066

**Table 2:** Relative luminescent intensity ratio (R) of the two transitions (^5^D_0_ → ^7^F_2_)/(^5^D_0_ → ^7^F_1_ for (50−x)MoO_3_:xWO_3_:25La_2_O_3_:25B_2_O_3_:3Eu_2_O_3_ (x = 0, 10, 20, 30, 40 and 50 mol%) glasses.

Glass Composition	Relative Intensity Ratio, R	Reference
50MoO_3_:25La_2_O_3_:25B_2_O_3_:3Eu_2_O_3_	7.09	Current work
40MoO_3_:10WO_3_:25La_2_O_3_:25B_2_O_3_:3Eu_2_O_3_	7.15	Current work
30MoO_3_:20WO_3_:25La_2_O_3_:25B_2_O_3_:3Eu_2_O_3_	7.19	Current work
20MoO_3_:30WO_3_:25La_2_O_3_:25B_2_O_3_:3Eu_2_O_3_	7.42	Current work
10MoO_3_:40WO_3_:25La_2_O_3_:25B_2_O_3_:3Eu_2_O_3_	7.63	Current work
50WO_3_:25La_2_O_3_:25B_2_O_3_:3Eu_2_O_3_	7.82	Current work
50ZnO:40B_2_O_3_:10WO_3_:xEu_2_O_3_ (0 ≤ x ≤ 10)	4.54÷5.77	[20]
50ZnO:40B_2_O_3_:5WO_3_:5Nb_2_O_5_:xEu_2_O_3_ (0 ≤ x ≤ 10)	5.09÷5.76	[45]
50ZnO:(50−x)B_2_O_3_:xNb_2_O_5_:0.5Eu_2_O_3_:, x = 0, 1, 3 and 5 mol%	4.31–5.16	[46]
50ZnO:(50−x)B_2_O_3_:0.5Eu_2_O_3_:xWO_3_, x = 0, 1, 3, 5	4.34–5.57	[21]
50ZnO:(49–x)B2O3:1Bi_2_O_3_:xWO_3_; x = 1, 5, 10	4.61–5.73	[47]
4ZnO:3B_2_O_3_ 0.5–2.5 mol % Eu_2_O_3_	2.74–3.94	[48]
60ZnO:20B_2_O_3_:(20−x)SiO_2_−xEu_2_O_3_ (x = 0 and 1)	3.166	[49]
15PbF_2_:25WO_3_:(60−x)TeO_2_:xEu_2_O_3_ x = 0.1, 0.5, 1.0 and 2.0 mol%	2.37–2.78	[16]
20PbO–5CaO–5ZnO–10LiF–59B_2_O_3_–1Eu_2_O	2.320	[50]
45SiO_2_−(20−x)PbF_2_−20K_2_O−5Na_2_O−10LiF−1.0Eu_2_O_3_	2.44	[51]
89.5B_2_O_3_–10Li_2_O–0.5Eu_2_O_3_	2.41	[52]
57GeO_2_–40K_2_O–3Eu_2_O_3_	3.70	[52]
73P2O5–25CaO–2Eu_2_O_3_	3.95	[52]
79TeO_2_−20Li_2_CO_3_−1Eu_2_O_3_	4.28	[53]
Eu^3+^:Y_2_O_3_	3.8–5.2	[54,55]
Eu^3+^:Y_2_O_2_S	6.45–6.62	[56]

## Data Availability

Data are contained within the article.
